# Versatile modeling and optimization of fed batch processes for the production of secreted heterologous proteins with *Pichia pastoris*

**DOI:** 10.1186/1475-2859-5-37

**Published:** 2006-12-11

**Authors:** Michael Maurer, Manfred Kühleitner, Brigitte Gasser, Diethard Mattanovich

**Affiliations:** 1 University of Natural Resources and Applied Life Sciences Vienna, Department of Biotechnology, Institute of Applied Microbiology, Vienna, Austria; 2 University of Natural Resources and Applied Life Sciences Vienna, Department of Integrative Biology, Institute of Mathematics, Vienna, Austria; 3 School of Bioengineering, University of Applied Sciences FH-Campus Vienna, Austria

## Abstract

**Background:**

Secretion of heterologous proteins depends both on biomass concentration and on the specific product secretion rate, which in turn is not constant at varying specific growth rates. As fed batch processes usually do not maintain a steady state throughout the feed phase, it is not trivial to model and optimize such a process by mathematical means.

**Results:**

We have developed a model for product accumulation in fed batch based on iterative calculation in Microsoft Excel spreadsheets, and used the Solver software to optimize the time course of the media feed in order to maximize the volumetric productivity. The optimum feed phase consisted of an exponential feed at maximum specific growth rate, followed by a phase with linearly increasing feed rate and consequently steadily decreasing specific growth rate. The latter phase could be modeled also by exact mathematical treatment by the calculus of variations, yielding the explicit shape of the growth function, however, with certain indeterminate parameters. To evaluate the latter, one needs a numerical optimum search algorithm. The explicit shape of the growth function provides additional evidence that the Excel model results in correct data. Experimental evaluation in two independent fed batch cultures resulted in a good correlation to the optimized model data, and a 2.2 fold improvement of the volumetric productivity.

**Conclusion:**

The advantages of the procedure we describe here are the ease of use and the flexibility, applying software familiar to every scientist and engineer, and rapid calculation which makes predictions extremely easy, so that many options can be tested *in silico *quickly. Additional options like further biological and technological constraints or different functions for specific productivity and biomass yield can easily be integrated.

## Background

Modeling of bioprocesses has been pursued since the 1970s, with the aim to rationally optimize processes. While the mathematical description of processes like growth and product formation have been fairly well achieved, it is still not routine practice to design biotechnological production processes based on model prediction. An especially difficult case in this respect is fed-batch as a dynamic system usually not reaching steady state. General attempts to model fed-batch processes have been described (for an overview see [1]). Based on these modeling approaches, optimization of fed batch processes has been attempted using Pontryagin's Maximum Principle [2,3], Green's Theorem [4], or Dynamic Programming [5]. These approaches are rather complex, and they did not find their way in routine application.

A typical case of fed-batch process is the production of recombinant proteins with microorganisms or mammalian cells. While the description of product concentration in the cell mass is rather straight forward (in the case of an intracellular product), it is more complex to predict the kinetics of a secreted product. A typical case for secretion systems are recombinant yeasts [6]. As the production of many proteins in yeasts is quite cost sensitive, it will be highly desirable to have a tool available that allows a simple yet reliable prediction of productivity, process time and product titers. Approaches to optimize fed batch processes for the methylotrophic yeast *Pichia pastoris *have been described [3,5]. The latter employ dynamic programming by dividing the total process time into a discrete number of intervals, and assigning a value of the specific growth rate *μ *selected from a discrete set of values. The major drawback of this approach is that the process time is fixed and not an issue of optimization. The algorithm used by this group is complex, and not readily available to others. Zhang and coworkers [3] present an approach based on Pontryagin's Maximum Principle. A general applicability seems hampered by the complex calculation, complicating a simple recalculation with modified data or calculation procedures.

With this work we aimed at the development of an optimization tool for fed batch processes using calculation tools available for every PC. MS Excel allows the approximation of a model by numerically solving equations describing the system, and the optimization of an objective function by modifying defined fields (the decision variables), while different constraints can be defined which have to be complied with. The calculation is based on the generalized reduced gradient method described in [7]. While the general concept of calculation is similar to the approaches above, the definition of the optimization objective – while obviously a crucial step – is not consistently resolved in the existing literature. The variable costs of a bioprocess correlate with the volumetric capacity of the required fermentation unit, and the process time this unit is required to produce a defined amount of the product [8]. Thus the volumetric productivity *Q*_*P *_is the most plausible target for optimization. At a given process time point *t*, *Q*_*P *_is defined as:

QP=PV⋅t     (1)
 MathType@MTEF@5@5@+=feaafiart1ev1aaatCvAUfKttLearuWrP9MDH5MBPbIqV92AaeXatLxBI9gBaebbnrfifHhDYfgasaacH8akY=wiFfYdH8Gipec8Eeeu0xXdbba9frFj0=OqFfea0dXdd9vqai=hGuQ8kuc9pgc9s8qqaq=dirpe0xb9q8qiLsFr0=vr0=vr0dc8meaabaqaciaacaGaaeqabaqabeGadaaakeaacqWGrbqudaWgaaWcbaGaemiuaafabeaakiabg2da9maalaaabaGaemiuaafabaGaemOvayLaeyyXICTaemiDaqhaaiaaxMaacaWLjaWaaeWaaeaacqaIXaqmaiaawIcacaGLPaaaaaa@3A22@

Expanding this concept to total manufacturing costs is feasible but depends on a profound and reliable cost calculation. As outlined below, *Q*_*P *_can be calculated from the specific growth rate *μ *and the specific production rate *q*_*P*_. *μ *should be one of the decision variables of the optimization (defining the feed rate profile to be developed), while *q*_*P *_depends on *μ*. The exact function, *q*_*P *_= *f*(*μ*), of this dependence for secreted recombinant proteins has been subject to discussion [9]. These authors provide some evidence that secreted protein productivity is saturated at high *μ*, but a clear experimental solution of this function and its biological basis has not been achieved yet. Zhang et al. approximate this relation by an empirical 3^rd ^order function [3], while Ohya and coworkers model it by a two step linear function [10]. Both groups base their model functions on rather few experimental data. To improve the accuracy of the model *q*_*P *_= *f*(*μ*), we examined the entire space of *μ *of a *P. pastoris *strain in chemostat cultures for the respective values of *q*_*P*_, as well as the observed biomass yield coefficient *Y'*_*XS *_in order to calculate the substrate needed for each increment of biomass increase. It has been discussed whether data derived from steady state are applicable to model transient situations as they usually occur in fed batch. The parameters determining the accuracy of a steady state model are the relaxation time constants of environmental changes and of biological processes based on the change in environment, which become critical at highly transient situations like a shift to growth limiting conditions at the end of batch [11]. However, substrate limited fed batch cannot be considered as highly transient, so that the steady state model should be applicable.

A *P. pastoris *strain expressing the Fab fragment of the anti-HIV antibody 2F5 [12] was employed as a model. As expression is based on the glyceraldehyde phosphate dehydrogenase (GAP) promoter, glucose is used as a substrate for growth. Modeling of the fed batch process and optimization of *Q*_*P *_was used to predict an optimal feed protocol, which was then evaluated experimentally. The model optimization was also solved analytically in order to prove the accuracy of the Excel approximation.

## Results and discussion

### Chemostat

**Figure 1:**
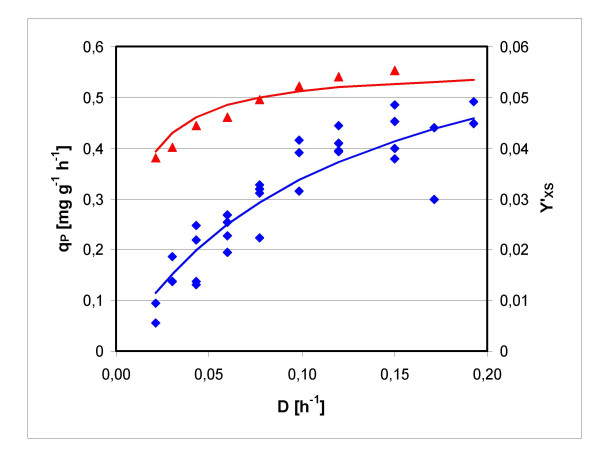
***q*_*P *_and *Y'*_*XS *_in chemostat cultures**. Specific product formation rate *q*_*P *_(blue diamonds) and observed biomass yield coefficient *Y'*_*XS *_(red triangles), as well as the respective approximations.

The 2F5 expression strain was cultivated in chemostat at dilution rates *D *between 0.0086 h^-1 ^and 0.2 h^-1^. Steady state samples were taken after 5 volume changes each. The setpoints were passed through once from high to low dilution rates, and once from low to high dilution rates. Specific production rates *q*_*P *_and observed biomass yield coefficients *Y'*_*XS *_are plotted against *D = μ *in figure 1. The constants of eq (22), describing *q*_*P*_, were derived by the method of least squares as *q*_*Pmax *_= 0.0735 mg g^-1 ^h^-1 ^and *k*_*q *_= 0.116 h^-1^. *Y*_*XS *_= 0.559 and *m*_*S *_= 0.0161 h^-1 ^were derived according to eq (26). The estimated standard deviation of *q*_*P *_is *s*_*q *_= 0.0048 mg g^-1 ^h^-1^, and that of *Y'*_*XS *_is *S*_*Y*' _= 0.023.

### Standard fed batch

**Figure 2:**
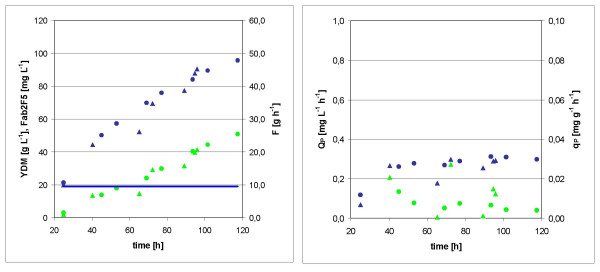
**Kinetics of standard fed batch cultures**. Values of two cultures (triangles and circles) are shown. A: biomass *x *(blue), product concentration *p *(green), and feed rate *F *(blue line) B: volumetric productivity *Q*_*P *_(blue) and specific product formation rate *q*_*P *_(green).

Two independent fed batch cultures using a standard protocol with constant feed rate [12] were performed. The final Fab titer was *p *= 46 mg L^-1^, and the final biomass concentration *x *= 96 g L^-1^, both at a total process time *t *= 117 h (92 h feed). Fig. 2A shows the development of these parameters over time, while *Q*_*P *_and *q*_*P *_are plotted in Fig. 2B. Apparently *Q*_*P *_has a maximum of 0.31 mg L^-1 ^h^-1 ^at t = 94 h (69 h feed).

### Optimized fed batch – model and experimental

**Figure 3:**
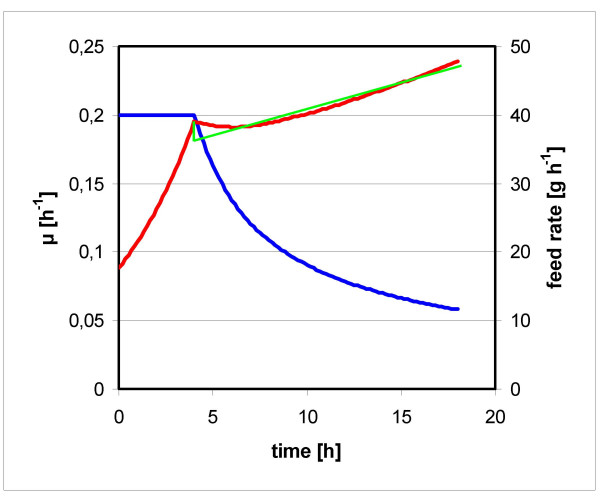
**Optimum time course of specific growth rate and medium feed**. *μ *(blue) and *F *(red) as obtained with the Solver. The approximated linearly increasing feed rate is shown in green.

Using the optimization algorithm described in Materials and Methods, a feed protocol leading to maximum volumetric productivity was determined. As the maximum final biomass concentration was set to 100 g L^-1^, and the feed medium was identical to the standard fed batch, almost the same biomass concentration and total feed volume was to be expected for both processes. Optimal *μ *and feed rate is plotted over time in Fig. 3. The feed starts with an exponential phase of 3.6 h, followed by a 16 h phase with more slowly increasing feed, which was approximated by a linearly increasing feed rate following the function

*F*_*L *_= 0.012 *g*·*h*^-2^·*t*_*L *_+ 30.672 *g*·*h*^-1 ^    (2)

**Figure 4:**
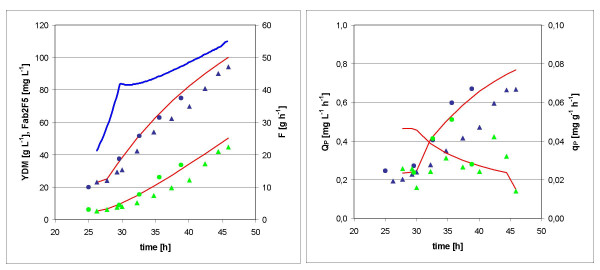
**Kinetics of optimized fed batch cultures**. Values of two cultures (triangles and circles) are shown. The model approximations are indicated by red lines. A: biomass *x *(blue), product concentration *p *(green), and feed rate (blue line) B: volumetric productivity *Q*_*P *_(blue) and specific product formation rate *q*_*P *_(green).

All values were calculated for a unit batch volume of 1 L and needed to be adjusted to the respective batch volume of 1.2 L. The modeled process was verified experimentally in two independent fed batch cultures. The resultant plots of biomass and product concentrations, as well as the predicted values, are displayed in Fig. 4A, while *Q*_*P *_and *q*_*P *_and their model prediction are shown in Fig. 4B. A final product concentration of 45 mg L^-1 ^was reached after 21 h feed at a biomass concentration of 94 g L^-1^. The maximum volumetric productivity *Q*_*P *_was 0.67 mg L^-1 ^h^-1 ^(slightly below the predicted value of 0.77 mg L^-1 ^h^-1^), which is a 2.2 fold improvement over the standard fed batch.

### Modeling of the standard fed batch

Using the same equations as for optimization we also attempted to model the standard fed batch process. However, the predicted values of biomass and product concentrations deviated significantly from the experimental data. Therefore we reconsidered the data source used to obtain the functions of *Y'*_*XS *_and *q*_*P *_based on *μ*. The obvious difference between the standard and optimized fed batch protocol is that the standard culture is performed at very low *μ *from 0.05 h^-1 ^decreasing to 0.005 h^-1^, while the optimized culture starts at *μ *= 0.2 h^-1^, decreasing to 0.05 h^-1^. As saturation functions like the Monod function are more susceptible to low values of the x-coordinate, and the majority of the data from chemostat were naturally obtained at higher *μ *values, we remodeled the function *q*_*P *_= *f*(*μ*) for low *μ *based on data derived from previous fed batch cultures. The best approximation in the range of *μ *≤ 0.05 h^-1 ^was a linear function

*q*_*P *_= 0.2051·*μ *+ 0.002     (3)

Similarly, *Y*_*XS *_and *m*_*S *_were remodeled in this range of *μ *≤ 0.05 h^-1^.

**Figure 5:**
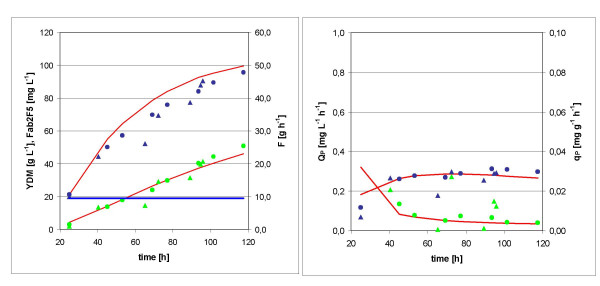
**Kinetics of standard fed batch cultures with adapted model approximations indicated by red lines**. Values of two cultures (triangles and circles) are shown. A: biomass *x *(blue), product concentration *p *(green), and feed rate (blue line) B: volumetric productivity *Q*_*P *_(blue) and specific product formation rate *q*_*P *_(green).

Based on these refined approximations, the predictions of biomass and product concentration, volumetric productivity and specific product formation rate fit well to the experimental values (Fig. 5).

Apparently the model based on chemostat data fits well at higher *μ*, while it needed adjustment at values below *μ *= 0.05 h^-1^. Most importantly, it was valid for the optimized feed protocol derived from the model, which led to a 2.2 fold increase of volumetric productivity. The sensitivity of the model to the accuracy of the function *q*_*P *_= *f*(*μ*) stresses the importance of an accurate experimental determination of *q*_*P *_both at low and high *μ*. Importantly, this function can be refined in future utilizing additional data from fed batch (and chemostat) cultures, so that the model acquires features of a self learning model.

### Analytic approach

The calculus of variations yields a method to derive an analytic formula (containing indeterminate parameters) for our optimization problem. Let *X *= *X*(*t*) be the amount of biomass, *P *= *P*(*t*) the amount of product and *μ *= *μ*(*t*) the specific growth rate of biomass at the point of time *t*. The process to be controlled is described by the following equations:

The growth of biomass is modeled by the equation

*X*'(*t*) = *μ*(*t*)·*X*(*t*)     (4)

with the initial value *X*(0) = *X*_0_.

The yield of product is modeled by the equation

*P*'(*t*) = *q*_*P*_(*t*)·*X*(*t*)     (5)

with a Monod like formula

qP(t)=qPmax⁡μ(t)kq+μ(t)     (6)
 MathType@MTEF@5@5@+=feaafiart1ev1aaatCvAUfKttLearuWrP9MDH5MBPbIqV92AaeXatLxBI9gBaebbnrfifHhDYfgasaacH8akY=wiFfYdH8Gipec8Eeeu0xXdbba9frFj0=OqFfea0dXdd9vqai=hGuQ8kuc9pgc9s8qqaq=dirpe0xb9q8qiLsFr0=vr0=vr0dc8meaabaqaciaacaGaaeqabaqabeGadaaakeaacqWGXbqCdaWgaaWcbaGaemiuaafabeaakiabcIcaOiabdsha0jabcMcaPiabg2da9iabdghaXnaaBaaaleaacqWGqbaucyGGTbqBcqGGHbqycqGG4baEaeqaaOWaaSaaaeaaiiGacqWF8oqBcqGGOaakcqWG0baDcqGGPaqkaeaacqWGRbWAdaWgaaWcbaGaemyCaehabeaakiabgUcaRiab=X7aTjabcIcaOiabdsha0jabcMcaPaaacaWLjaGaaCzcamaabmaabaGaeGOnaydacaGLOaGaayzkaaaaaa@4BFC@

and the initial value *P*(0) = *P*_0_.

In a first step we maximize the cumulative yield of product in a fixed time interval [0, T]. Therefore, 1T
 MathType@MTEF@5@5@+=feaafiart1ev1aaatCvAUfKttLearuWrP9MDH5MBPbIqV92AaeXatLxBI9gBaebbnrfifHhDYfgasaacH8akY=wiFfYdH8Gipec8Eeeu0xXdbba9frFj0=OqFfea0dXdd9vqai=hGuQ8kuc9pgc9s8qqaq=dirpe0xb9q8qiLsFr0=vr0=vr0dc8meaabaqaciaacaGaaeqabaqabeGadaaakeaadaWcaaqaaiabigdaXaqaaiabbsfaubaaaaa@2EDB@P(T) → Max is equivalent to P(T) → Max. We therefore consider

P(t)=∫0TP′(t)⋅dt=qPmax⁡∫0Tμ(t)kq+μ(t)X(t)⋅dt     (7)
MathType@MTEF@5@5@+=feaafiart1ev1aaatCvAUfKttLearuWrP9MDH5MBPbIqV92AaeXatLxBI9gBaebbnrfifHhDYfgasaacH8akY=wiFfYdH8Gipec8Eeeu0xXdbba9frFj0=OqFfea0dXdd9vqai=hGuQ8kuc9pgc9s8qqaq=dirpe0xb9q8qiLsFr0=vr0=vr0dc8meaabaqaciaacaGaaeqabaqabeGadaaakeaacqWGqbaucqGGOaakcqWG0baDcqGGPaqkcqGH9aqpdaWdXbqaaiqbdcfaqzaafaGaeiikaGIaemiDaqNaeiykaKIaeyyXICTaemizaqMaemiDaqhaleaacqaIWaamaeaacqWGubava0Gaey4kIipakiabg2da9iabdghaXnaaBaaaleaacqWGqbaucyGGTbqBcqGGHbqycqGG4baEaeqaaOWaa8qCaeaadaWcaaqaaGGaciab=X7aTjabcIcaOiabdsha0jabcMcaPaqaaiabdUgaRnaaBaaaleaacqWGXbqCaeqaaOGaey4kaSIae8hVd0MaeiikaGIaemiDaqNaeiykaKcaaaWcbaGaeGimaadabaGaemivaqfaniabgUIiYdGccqWGybawcqGGOaakcqWG0baDcqGGPaqkcqGHflY1cqWGKbazcqWG0baDcaWLjaGaaCzcamaabmaabaGaeG4naCdacaGLOaGaayzkaaaaaa@6753@

by formulas (5) and (6). Inserting *X*'(*t*)/*X*(*t*) for *μ*(*t*) results in maximizing the integral

I(X)=∫0TX(t)⋅X′(t)kq⋅X(t)+X′(t)dt     (8)
MathType@MTEF@5@5@+=feaafiart1ev1aaatCvAUfKttLearuWrP9MDH5MBPbIqV92AaeXatLxBI9gBaebbnrfifHhDYfgasaacH8akY=wiFfYdH8Gipec8Eeeu0xXdbba9frFj0=OqFfea0dXdd9vqai=hGuQ8kuc9pgc9s8qqaq=dirpe0xb9q8qiLsFr0=vr0=vr0dc8meaabaqaciaacaGaaeqabaqabeGadaaakeaacqWGjbqscqGGOaakcqWGybawcqGGPaqkcqGH9aqpdaWdXbqaamaalaaabaGaemiwaGLaeiikaGIaemiDaqNaeiykaKIaeyyXICTafmiwaGLbauaacqGGOaakcqWG0baDcqGGPaqkaeaacqWGRbWAdaWgaaWcbaGaemyCaehabeaakiabgwSixlabdIfayjabcIcaOiabdsha0jabcMcaPiabgUcaRiqbdIfayzaafaGaeiikaGIaemiDaqNaeiykaKcaaaWcbaGaeGimaadabaGaemivaqfaniabgUIiYdGccqWGKbazcqWG0baDcaWLjaGaaCzcamaabmaabaGaeGioaGdacaGLOaGaayzkaaaaaa@56E5@

This integral has an extremum only if the Euler-Lagrange differential equation

∂F∂x−ddt∂F∂X′=0     (9)
 MathType@MTEF@5@5@+=feaafiart1ev1aaatCvAUfKttLearuWrP9MDH5MBPbIqV92AaeXatLxBI9gBaebbnrfifHhDYfgasaacH8akY=wiFfYdH8Gipec8Eeeu0xXdbba9frFj0=OqFfea0dXdd9vqai=hGuQ8kuc9pgc9s8qqaq=dirpe0xb9q8qiLsFr0=vr0=vr0dc8meaabaqaciaacaGaaeqabaqabeGadaaakeaadaWcaaqaaiabgkGi2kabdAeagbqaaiabgkGi2kabdIha4baacqGHsisldaWcaaqaaiabdsgaKbqaaiabdsgaKjabdsha0baadaWcaaqaaiabgkGi2kabdAeagbqaaiabgkGi2kqbdIfayzaafaaaaiabg2da9iabicdaWiaaxMaacaWLjaWaaeWaaeaacqaI5aqoaiaawIcacaGLPaaaaaa@421D@

is satisfied with F=F(X,X′)=X⋅X′k⋅X+X′     (10)
 MathType@MTEF@5@5@+=feaafiart1ev1aaatCvAUfKttLearuWrP9MDH5MBPbIqV92AaeXatLxBI9gBaebbnrfifHhDYfgasaacH8akY=wiFfYdH8Gipec8Eeeu0xXdbba9frFj0=OqFfea0dXdd9vqai=hGuQ8kuc9pgc9s8qqaq=dirpe0xb9q8qiLsFr0=vr0=vr0dc8meaabaqaciaacaGaaeqabaqabeGadaaakeaacqqGPbqAcqqGZbWCcqqGGaaicqqGZbWCcqqGHbqycqqG0baDcqqGPbqAcqqGZbWCcqqGMbGzcqqGPbqAcqqGLbqzcqqGKbazcqqGGaaicqqG3bWDcqqGPbqAcqqG0baDcqqGObaAcqqGGaaicqWGgbGrcqGH9aqpcqWGgbGrcqGGOaakcqWGybawcqGGSaalcuWGybawgaqbaiabcMcaPiabg2da9maalaaabaGaemiwaGLaeyyXICTafmiwaGLbauaaaeaacqWGRbWAcqGHflY1cqWGybawcqGHRaWkcuWGybawgaqbaaaacaWLjaGaaCzcamaabmaabaGaeGymaeJaeGimaadacaGLOaGaayzkaaaaaa@5D64@

Evaluating the Euler Lagrange equation (9) yields

X′2(kq⋅X+X′)2−ddtkq⋅X2(kq⋅X+X′)2=0     (11)
 MathType@MTEF@5@5@+=feaafiart1ev1aaatCvAUfKttLearuWrP9MDH5MBPbIqV92AaeXatLxBI9gBaebbnrfifHhDYfgasaacH8akY=wiFfYdH8Gipec8Eeeu0xXdbba9frFj0=OqFfea0dXdd9vqai=hGuQ8kuc9pgc9s8qqaq=dirpe0xb9q8qiLsFr0=vr0=vr0dc8meaabaqaciaacaGaaeqabaqabeGadaaakeaadaWcaaqaaiqbdIfayzaafaWaaWbaaSqabeaacqaIYaGmaaaakeaacqGGOaakcqWGRbWAdaWgaaWcbaGaemyCaehabeaakiabgwSixlabdIfayjabgUcaRiqbdIfayzaafaGaeiykaKYaaWbaaSqabeaacqaIYaGmaaaaaOGaeyOeI0YaaSaaaeaacqWGKbazaeaacqWGKbazcqWG0baDaaWaaSaaaeaacqWGRbWAdaWgaaWcbaGaemyCaehabeaakiabgwSixlabdIfaynaaCaaaleqabaGaeGOmaidaaaGcbaGaeiikaGIaem4AaS2aaSbaaSqaaiabdghaXbqabaGccqGHflY1cqWGybawcqGHRaWkcuWGybawgaqbaiabcMcaPmaaCaaaleqabaGaeGOmaidaaaaakiabg2da9iabicdaWiaaxMaacaWLjaWaaeWaaeaacqaIXaqmcqaIXaqmaiaawIcacaGLPaaaaaa@59A1@

Inserting *μ*(*t*)·*X*(*t*) for *X*'(*t*) (from eq. 4) yields

μ2(kq+μ)2−ddtkq(kq+μ)2=0     (12)
 MathType@MTEF@5@5@+=feaafiart1ev1aaatCvAUfKttLearuWrP9MDH5MBPbIqV92AaeXatLxBI9gBaebbnrfifHhDYfgasaacH8akY=wiFfYdH8Gipec8Eeeu0xXdbba9frFj0=OqFfea0dXdd9vqai=hGuQ8kuc9pgc9s8qqaq=dirpe0xb9q8qiLsFr0=vr0=vr0dc8meaabaqaciaacaGaaeqabaqabeGadaaakeaadaWcaaqaaGGaciab=X7aTnaaCaaaleqabaGaeGOmaidaaaGcbaGaeiikaGIaem4AaS2aaSbaaSqaaiabdghaXbqabaGccqGHRaWkcqWF8oqBcqGGPaqkdaahaaWcbeqaaiabikdaYaaaaaGccqGHsisldaWcaaqaaiabdsgaKbqaaiabdsgaKjabdsha0baadaWcaaqaaiabdUgaRnaaBaaaleaacqWGXbqCaeqaaaGcbaGaeiikaGIaem4AaS2aaSbaaSqaaiabdghaXbqabaGccqGHRaWkcqWF8oqBcqGGPaqkdaahaaWcbeqaaiabikdaYaaaaaGccqGH9aqpcqaIWaamcaWLjaGaaCzcamaabmaabaGaeGymaeJaeGOmaidacaGLOaGaayzkaaaaaa@4F41@

Calculating the differential and reducing to a common denominator gives

2·*k*_*q*_·*μ'*(*t*) + *μ*^3^(*t*) + *k*_*q*_·*μ*^2^(*t*) = 0     (13)

We solve this differential equation and get the following equation for *μ*(*t*):

t+c=2μ+2kqln⁡μkq+μ     (14)
 MathType@MTEF@5@5@+=feaafiart1ev1aaatCvAUfKttLearuWrP9MDH5MBPbIqV92AaeXatLxBI9gBaebbnrfifHhDYfgasaacH8akY=wiFfYdH8Gipec8Eeeu0xXdbba9frFj0=OqFfea0dXdd9vqai=hGuQ8kuc9pgc9s8qqaq=dirpe0xb9q8qiLsFr0=vr0=vr0dc8meaabaqaciaacaGaaeqabaqabeGadaaakeaacqWG0baDcqGHRaWkcqWGJbWycqGH9aqpdaWcaaqaaiabikdaYaqaaGGaciab=X7aTbaacqGHRaWkdaWcaaqaaiabikdaYaqaaiabdUgaRnaaBaaaleaacqWGXbqCaeqaaaaakiGbcYgaSjabc6gaUnaalaaabaGae8hVd0gabaGaem4AaS2aaSbaaSqaaiabdghaXbqabaGccqGHRaWkcqWF8oqBaaGaaCzcaiaaxMaadaqadaqaaiabigdaXiabisda0aGaayjkaiaawMcaaaaa@47C4@

**Figure 6:**
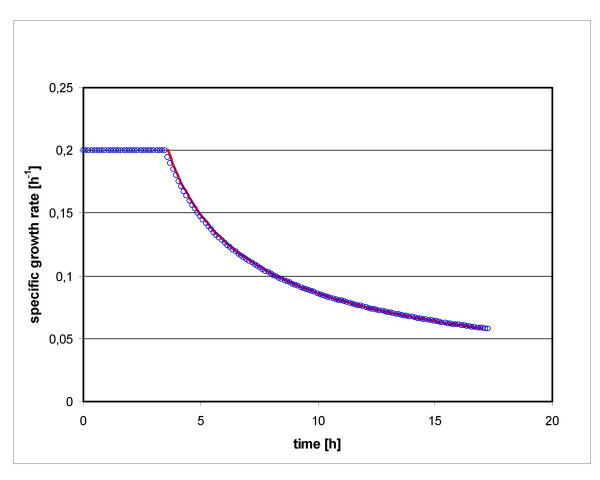
**Optimized time course of specific growth rate**. Overlay of the solution obtained by Solver (blue circles) and analytic solution (red line).

Eq (14) defines only a necessary condition for the optimum trajectory of *μ *over time, with indetermined parameter *c *and indetermined optimal value for the total feed time *T*. Here the parameter *c *and the optimal value for the total feed period *T *depend on the constraints *μ*_min_, *μ*_max _and *q*_*P*max_. Since we know a numerical optimal solution for the growth function, we calculate the value *c *by fitting the analytic curve (eq 14) to the optimal solution calculated by Excel with the method of least squares. We get as numeric value *c *= -1.49967812 h. Figure 6 shows the excellent correspondence of the analytic solution and the solution calculated by the Excel Solver. This proves that the Solver solution obeys the necessary condition of the maximization problem, as defined by the Euler-Lagrange equation.

## Conclusion

We have developed a modeling and optimization algorithm for fed batch cultures of secreted products based on MS Excel. The validity of this iterative calculation, which is highly flexible and versatile, was proven by analytic solution of the equations forming the basis of the fed batch model. While the analytic solution fits exactly to the phase of decreasing specific growth rate of the Excel Solver solution, it is not possible to calculate the duration of the initial *μ*_max _phase. As the optimum feed profiles obviously consist of an exponential phase followed by a phase of steadily decreasing *μ*, the analytic approach could only serve as evidence of the correct solution of the optimization problem obtained with Excel Solver. Both the Euler-Lagrange approach used here and Pontryagin's Maximum Principle depend on data fitting to obtain a numeric solution. Given the perfect match of the two approaches presented here, we consider it much more straight forward to apply a numeric data fitting approach directly to the equations of growth and product formation.

The advantages of the procedure we describe here are the ease of use, applying software familiar to every scientist and engineer, and rapid calculation which makes predictions extremely easy, so that many options can be tested *in silico *quickly. Additional options like further biological and technological constraints or different functions for specific productivity and biomass yield can easily be integrated.

We could prove that the experimental data basis for the functions behind the algorithm is very important. Different to previous work this was taken into account, and especially the sensitivity at very low specific growth rates needs to be highlighted.

The Excel file containing the model and optimization procedure is provided as accompanying file [see additional file 1].

## Materials and methods

Unless stated otherwise, all chemicals were purchased from Merck Eurolab and all antisera were from Sigma.

### Strain

A *P. pastoris *strain X33 (wild type strain) expressing extracellularly the Fab fragment of the anti-HIV antibody 2F5 under control of the GAP promoter was used in this study. The development of this strain has been described elsewhere [12]. A cell bank of the strain was prepared, divided in 1.8 mL aliquots and stored at -80°C.

### Fermentation

A shake flask containing 100 mL of YPG medium (per liter: 10 g yeast extract, 10 g peptone, 10 g glycerol) was inoculated with one cryovial from the *P. pastoris *cell bank, and incubated at 28°C for approximately 24 hours and agitated at 180 rpm.

This culture was used to inoculate the starting volume in the bioreactor to a starting optical density (OD_600_) of 1.0. Depending on the operation mode the starting volume was either 1.2 L for fed batch or 1.4 L for chemostat process.

Fermentations were carried out in a 2.0 L working volume bioreactor (MBR; Wetzikon, Switzerland) with a computer based process control (ISE; Vienna, Austria). Fermentation temperature was controlled at 25°C, pH was controlled at 5.0 with addition of 25% ammonium hydroxide and the dissolved oxygen concentration was maintained above 20% saturation by controlling the stirrer speed between 600 and 1200 rpm, whereas the airflow was kept constant at 100 L h^-1^.

The media were as follows:

Batch medium contained per liter:

2.0 g citric acid, 12.4 g (NH_4_)_2_HPO_4_, 0.022 g CaCl_2_·2H_2_O, 0.9 g KCl, 0.5 g MgSO_4_·7H_2_O, 40 g glycerol, 4.6 ml PTM_1 _trace salts stock solution. The pH was set to 5.0 with 25% HCl.

Glucose fed batch solution contained per liter:

550 g glucose·1H_2_O, 10 g KCl, 6.45 g MgSO_4_·7H_2_O, 0.35 g CaCl_2_·2H_2_O and 12 ml PTM_1 _trace salts stock solution.

Chemostat medium contained per liter:

55 g glucose·1H_2_O, 2.5 g KCl, 1.0 g MgSO_4_·7H_2_O, 0.035 g CaCl_2_·2H_2_O, 21.8 g (NH_4_)_2_HPO_4 _and 2.4 ml PTM_1 _trace salts stock solution, furthermore the pH was set to 5.0 with 25% HCl.

PTM_1_ trace salts stock solution contained per liter:

6.0 g CuSO_4_·5H_2_O, 0.08 g NaI, 3.0 g MnSO_4_· H_2_O, 0.2 g Na_2_MoO_4_·2H_2_O, 0.02 g H_3_BO_3_, 0.5 g CoCl_2_, 20.0 g ZnCl_2_, 65.0 g FeSO_4_·7H_2_O, 0.2 g biotin and 5.0 ml H_2_SO_4 _(95%–98%). All chemicals for PTM_1 _trace salts stock solution were from Riedel-de Haën (Seelze, Germany), except for biotin (Sigma, St. Louis, MO, USA), and H_2_SO_4 _(Merck Eurolab).

After approximately 24 hours the batch was finished and – depending on the fermentation strategy – the feed and if required the harvest was started.

The continuous fermentation was initiated at a *D *= 0.15 h^-1 ^and performed at least for 5 resident times *τ *to reach steady state conditions.

τ=1D=VF     (15)
 MathType@MTEF@5@5@+=feaafiart1ev1aaatCvAUfKttLearuWrP9MDH5MBPbIqV92AaeXatLxBI9gBaebbnrfifHhDYfgasaacH8akY=wiFfYdH8Gipec8Eeeu0xXdbba9frFj0=OqFfea0dXdd9vqai=hGuQ8kuc9pgc9s8qqaq=dirpe0xb9q8qiLsFr0=vr0=vr0dc8meaabaqaciaacaGaaeqabaqabeGadaaakeaaiiGacqWFepaDcqGH9aqpdaWcaaqaaiabigdaXaqaaiabdseaebaacqGH9aqpdaWcaaqaaiabdAfawbqaaiabdAeagbaacaWLjaGaaCzcamaabmaabaGaeGymaeJaeGynaudacaGLOaGaayzkaaaaaa@39A4@

Then the dilution rate was decreased stepwise, always achieving steady state conditions before the next change of the dilution rate. At *D *= 0.0086 h^-1 ^the procedure was reversed and the dilution rate was increased stepwise up to the critical dilution rate *D*_crit _= 0.2 h^-1^. Samples were taken after 3 and 5 *τ *and analyzed as described below.

The standard fermentation strategy was a fed batch with a constant feed, this means that the batch phase was followed by the glucose fed batch with a feed rate *F *= 8.925 g h^-1^. The fermentations were terminated at appr. t = 120 h. Samples were taken frequently and processed as described below.

The optimized fermentation strategy consists of different phases to perform the calculated growth kinetic. The batch phase was followed by an exponential feed phase with a growth rate of 0.2 for 3.6 hours, followed by a linearly increasing feed rate calculated by equation (16), where k = 0.0144 g h^-2 ^and d = 36.8064 g h^-1 ^for 16.0 hours.

*F*_*L *_= *k*·*t*_*L *_+ *d *    (16)

### Analytical methods

#### Optical density

The samples were diluted in ddH_2_O up to 1:500 to measure the OD at 600 nm.

#### Biomass determination

2 × 5 ml culture were centrifuged and the supernatants frozen for further analysis. The pellets were resuspended in ddH_2_O, recentrifuged, and the pellets again resuspended in ddH_2_O, transferred to a weighed beaker, dried at 105°C until constant weight.

#### Product quantification (ELISA)

To determine the Fab content, 96 well microtiter plates (MaxiSorb, Nunc, Denmark) were coated with anti-hIgG (Fab specific) overnight at RT (1:1000 in PBS, pH 7.4), before serially diluted supernatants of *P. pastoris *cultures secreting 2F5 Fab (starting with a 1:100 dilution in PBS) were applied and incubated for 2 h at RT. Fab of normal IgG (Nordic) was used as a standard protein at a starting concentration of 200 ng/ml. After each incubation step the plates were washed four times with PBS containing 1% Tween 20 adjusted to pH 7.4. 100 *μ*l of anti-kappa light chain – AP conjugate as secondary antibody (1:1000 in PBS/Tween + 2% BSA) were added to each well, and incubated for 1 h at RT. After washing, the plates were stained with pNPP (1 mg/ml p-nitrophenyl phosphate in coating buffer, 0.1 N Na_2_CO_3_/NaHCO_3_; pH 9.6) and read at 405 nm (reference wavelength 620 nm).

### Method of calculation

#### 1. Setup of calculations

We divide the total feed period in equal intervals [*t*_*n*_, *t*_*n*+1_] (1 ≤ *n *≤ *N*) of length *dt*. Therefore,

*t*_*n*+1 _= *t*_*n *_+ *dt *    (17)

We start with an initial value *dt *= 1 [h]. The best value for *dt *is determined within the optimization process.

At every point of time *t*_*n *_we denote by *X*_*n *_= *X*(*t*_*n*_) the amount of biomass and by *P*_*n *_= *P*(*t*_*n*_) the amount of product in the bioreactor. At the beginning of the fed-batch process the initial values are *X*(0) = *X*_0 _and *P*(0) = *P*_0_, as achieved at the end of the batch phase.

First we have to describe the growth of the biomass. We use the simplest model, the exponential growth model,

dXdt=μ⋅X     (18)
 MathType@MTEF@5@5@+=feaafiart1ev1aaatCvAUfKttLearuWrP9MDH5MBPbIqV92AaeXatLxBI9gBaebbnrfifHhDYfgasaacH8akY=wiFfYdH8Gipec8Eeeu0xXdbba9frFj0=OqFfea0dXdd9vqai=hGuQ8kuc9pgc9s8qqaq=dirpe0xb9q8qiLsFr0=vr0=vr0dc8meaabaqaciaacaGaaeqabaqabeGadaaakeaadaWcaaqaaiabdsgaKjabdIfaybqaaiabdsgaKjabdsha0baacqGH9aqpiiGacqWF8oqBcqGHflY1cqWGybawcaWLjaGaaCzcamaabmaabaGaeGymaeJaeGioaGdacaGLOaGaayzkaaaaaa@3D09@

Since the specific growth rate *μ *of the biomass depends on time, we calculate (eq. 18) in discrete time steps

Xn+1=Xn⋅eμndt     (19)
 MathType@MTEF@5@5@+=feaafiart1ev1aaatCvAUfKttLearuWrP9MDH5MBPbIqV92AaeXatLxBI9gBaebbnrfifHhDYfgasaacH8akY=wiFfYdH8Gipec8Eeeu0xXdbba9frFj0=OqFfea0dXdd9vqai=hGuQ8kuc9pgc9s8qqaq=dirpe0xb9q8qiLsFr0=vr0=vr0dc8meaabaqaciaacaGaaeqabaqabeGadaaakeaacqWGybawdaWgaaWcbaGaemOBa4Maey4kaSIaeGymaedabeaakiabg2da9iabdIfaynaaBaaaleaacqWGUbGBaeqaaOGaeyyXICTaemyzau2aaWbaaSqabeaaiiGacqWF8oqBdaWgaaadbaGaemOBa4gabeaaliabdsgaKjabdsha0baakiaaxMaacaWLjaWaaeWaaeaacqaIXaqmcqaI5aqoaiaawIcacaGLPaaaaaa@43D9@

where *μ*_*n *_is the specific growth rate during the interval [*t*_*n*_, *t*_*n*+1_]. The initial values for *μ*_*n *_are chosen arbitrarily, for instance *μ*_*n *_≡ *μ*_max_. The optimal values for all of the *μ*_*n*_'s are determined within the optimization process.

Second we have to describe the accumulation of the product. We simply calculate the total product yield during the interval [*t*_*n*_, *t*_*n*+1_] by the following formula

*P*_*n*+1 _= *P*_*n *_+ *dP*_*n *_    (20)

with

*dP*_*n *_= q_*Pn*_· *X*_*n*_·*dt *    (21)

The relationship between the specific rate *q*_*P *_of product formation and the specific growth rate *μ *was experimentally determined in chemostat cultures. The dependence of *q*_*P *_on *μ *was described analogous to Monod equation:

qPn=qPmax⁡⋅μnkq+μn     (22)
 MathType@MTEF@5@5@+=feaafiart1ev1aaatCvAUfKttLearuWrP9MDH5MBPbIqV92AaeXatLxBI9gBaebbnrfifHhDYfgasaacH8akY=wiFfYdH8Gipec8Eeeu0xXdbba9frFj0=OqFfea0dXdd9vqai=hGuQ8kuc9pgc9s8qqaq=dirpe0xb9q8qiLsFr0=vr0=vr0dc8meaabaqaciaacaGaaeqabaqabeGadaaakeaacqWGXbqCdaWgaaWcbaGaemiuaaLaemOBa4gabeaakiabg2da9iabdghaXnaaBaaaleaacqWGqbaucyGGTbqBcqGGHbqycqGG4baEaeqaaOGaeyyXIC9aaSaaaeaaiiGacqWF8oqBdaWgaaWcbaGaemOBa4gabeaaaOqaaiabdUgaRnaaBaaaleaacqWGXbqCaeqaaOGaey4kaSIae8hVd02aaSbaaSqaaiabd6gaUbqabaaaaOGaaCzcaiaaxMaadaqadaqaaiabikdaYiabikdaYaGaayjkaiaawMcaaaaa@4A62@

The values for *q*_*Pmax *_and *k*_*q *_are derived from the experimental data by the method of least squares, i.e. the parameters *q*_*P*max _and *k*_*q *_are chosen that the sum of the deviations from the experimental data squared is minimal.

Next we have to calculate the amount of substrate *dS *which we must feed in the time interval [*t*_*n*_, *t*_*n*+1_]. To do this, let *S*_*n *_be the amount of substrate added to the bioreactor until the time point *t*_*n*_. Then the substrate consumption rate depends on the amount and on the increase of biomass, i.e.

dSdt=−(mS⋅X+1YXSdXdt)     (23)
 MathType@MTEF@5@5@+=feaafiart1ev1aaatCvAUfKttLearuWrP9MDH5MBPbIqV92AaeXatLxBI9gBaebbnrfifHhDYfgasaacH8akY=wiFfYdH8Gipec8Eeeu0xXdbba9frFj0=OqFfea0dXdd9vqai=hGuQ8kuc9pgc9s8qqaq=dirpe0xb9q8qiLsFr0=vr0=vr0dc8meaabaqaciaacaGaaeqabaqabeGadaaakeaadaWcaaqaaiabdsgaKjabdofatbqaaiabdsgaKjabdsha0baacqGH9aqpcqGHsislcqGGOaakcqWGTbqBdaWgaaWcbaGaem4uamfabeaakiabgwSixlabdIfayjabgUcaRmaalaaabaGaeGymaedabaGaemywaK1aaSbaaSqaaiabdIfayjabdofatbqabaaaaOWaaSaaaeaacqWGKbazcqWGybawaeaacqWGKbazcqWG0baDaaGaeiykaKIaaCzcaiaaxMaadaqadaqaaiabikdaYiabiodaZaGaayjkaiaawMcaaaaa@4BB8@

where *m*_*S *_is the maintenance coefficient and *Y*_*XS *_is the true yield coefficient of biomass from substrate. Inserting formula (18) in (23) the amount of substrate feed in the interval [*t*_*n*_, *t*_*n*+1_] calculates as

dSn=(μnYXS+mS)⋅Xn⋅dt     (24)
 MathType@MTEF@5@5@+=feaafiart1ev1aaatCvAUfKttLearuWrP9MDH5MBPbIqV92AaeXatLxBI9gBaebbnrfifHhDYfgasaacH8akY=wiFfYdH8Gipec8Eeeu0xXdbba9frFj0=OqFfea0dXdd9vqai=hGuQ8kuc9pgc9s8qqaq=dirpe0xb9q8qiLsFr0=vr0=vr0dc8meaabaqaciaacaGaaeqabaqabeGadaaakeaacqWGKbazcqWGtbWudaWgaaWcbaGaemOBa4gabeaakiabg2da9iabcIcaOmaalaaabaacciGae8hVd02aaSbaaSqaaiabd6gaUbqabaaakeaacqWGzbqwdaWgaaWcbaGaemiwaGLaem4uamfabeaaaaGccqGHRaWkcqWGTbqBdaWgaaWcbaGaem4uamfabeaakiabcMcaPiabgwSixlabdIfaynaaBaaaleaacqWGUbGBaeqaaOGaeyyXICTaemizaqMaemiDaqNaaCzcaiaaxMaadaqadaqaaiabikdaYiabisda0aGaayjkaiaawMcaaaaa@4D49@

To calculate the parameters *Y*_*XS *_and *m*_*S *_from experimental data of chemostat cultures by the method of least squares, we use the observed biomass yield coefficient *Y*'_*XS *_depending on the specific growth rate *μ*. This is done by *dX *= -*Y*'_*XS*_·*dS *and inserting formula (18) and the formula for the whole substrate consumption which implies

Y′XS=μμYXS+mS     (25)
 MathType@MTEF@5@5@+=feaafiart1ev1aaatCvAUfKttLearuWrP9MDH5MBPbIqV92AaeXatLxBI9gBaebbnrfifHhDYfgasaacH8akY=wiFfYdH8Gipec8Eeeu0xXdbba9frFj0=OqFfea0dXdd9vqai=hGuQ8kuc9pgc9s8qqaq=dirpe0xb9q8qiLsFr0=vr0=vr0dc8meaabaqaciaacaGaaeqabaqabeGadaaakeaacuWGzbqwgaqbamaaBaaaleaacqWGybawcqWGtbWuaeqaaOGaeyypa0ZaaSaaaeaaiiGacqWF8oqBaeaadaWcaaqaaiab=X7aTbqaaiabdMfaznaaBaaaleaacqWGybawcqWGtbWuaeqaaaaakiabgUcaRiabd2gaTnaaBaaaleaacqWGtbWuaeqaaaaakiaaxMaacaWLjaWaaeWaaeaacqaIYaGmcqaI1aqnaiaawIcacaGLPaaaaaa@415F@

Formula (25) can be transformed to

1Y′XS=1YXS+mSμ     (26)
 MathType@MTEF@5@5@+=feaafiart1ev1aaatCvAUfKttLearuWrP9MDH5MBPbIqV92AaeXatLxBI9gBaebbnrfifHhDYfgasaacH8akY=wiFfYdH8Gipec8Eeeu0xXdbba9frFj0=OqFfea0dXdd9vqai=hGuQ8kuc9pgc9s8qqaq=dirpe0xb9q8qiLsFr0=vr0=vr0dc8meaabaqaciaacaGaaeqabaqabeGadaaakeaadaWcaaqaaiabigdaXaqaaiqbdMfazzaafaWaaSbaaSqaaiabdIfayjabdofatbqabaaaaOGaeyypa0ZaaSaaaeaacqaIXaqmaeaacqWGzbqwdaWgaaWcbaGaemiwaGLaem4uamfabeaaaaGccqGHRaWkdaWcaaqaaiabd2gaTnaaBaaaleaacqWGtbWuaeqaaaGcbaacciGae8hVd0gaaiaaxMaacaWLjaWaaeWaaeaacqaIYaGmcqaI2aGnaiaawIcacaGLPaaaaaa@41A0@

From this double reciprocal plot *Y*_*XS *_and *m*_*S *_were determined by linear regression.

Last but not least we need the total volume for the calculation of the volumetric productivity. The model process starts with a batch volume of *V*_0 _= 1 L. The total volume at each time interval is then

Vn+1=Vn+dSnsf⋅ρf⋅1000     (27)
 MathType@MTEF@5@5@+=feaafiart1ev1aaatCvAUfKttLearuWrP9MDH5MBPbIqV92AaeXatLxBI9gBaebbnrfifHhDYfgasaacH8akY=wiFfYdH8Gipec8Eeeu0xXdbba9frFj0=OqFfea0dXdd9vqai=hGuQ8kuc9pgc9s8qqaq=dirpe0xb9q8qiLsFr0=vr0=vr0dc8meaabaqaciaacaGaaeqabaqabeGadaaakeaacqWGwbGvdaWgaaWcbaGaemOBa4Maey4kaSIaeGymaedabeaakiabg2da9iabdAfawnaaBaaaleaacqWGUbGBaeqaaOGaey4kaSYaaSaaaeaacqWGKbazcqWGtbWudaWgaaWcbaGaemOBa4gabeaaaOqaaiabdohaZnaaBaaaleaacqWGMbGzaeqaaOGaeyyXICncciGae8xWdi3aaSbaaSqaaiabdAgaMbqabaGccqGHflY1cqaIXaqmcqaIWaamcqaIWaamcqaIWaamaaGaaCzcaiaaxMaadaqadaqaaiabikdaYiabiEda3aGaayjkaiaawMcaaaaa@4D86@

with the substrate concentration in the feed medium s_*f *_and the density of the feed medium *ρ*_*f*_. Due to the high biomass concentrations achieved in *P. pastoris *fermentations, the cells occupy a significant fraction of the total volume, while the product is secreted to the liquid phase, the culture supernatant. In order to calculate the product concentration, the available liquid volume *V*_*l *_is calculated at each time interval with the specific volume of wet biomass, which is derived from dry biomass as the specific volume per dry biomass *ν*_YDM _= 0.0033 L g^-1^.

*V*_ln _= *V*_*n *_- *X*_*n*_·*ν*_*YDM *_    (28)

Finally, we calculate the biomass and the product concentrations. The product concentration *p *at the time point *t*_*n *_is calculated as

pn=PnVln⁡     (29)
 MathType@MTEF@5@5@+=feaafiart1ev1aaatCvAUfKttLearuWrP9MDH5MBPbIqV92AaeXatLxBI9gBaebbnrfifHhDYfgasaacH8akY=wiFfYdH8Gipec8Eeeu0xXdbba9frFj0=OqFfea0dXdd9vqai=hGuQ8kuc9pgc9s8qqaq=dirpe0xb9q8qiLsFr0=vr0=vr0dc8meaabaqaciaacaGaaeqabaqabeGadaaakeaacqWGWbaCdaWgaaWcbaGaemOBa4gabeaakiabg2da9maalaaabaGaemiuaa1aaSbaaSqaaiabd6gaUbqabaaakeaacqWGwbGvdaWgaaWcbaGagiiBaWMaeiOBa4gabeaaaaGccaWLjaGaaCzcamaabmaabaGaeGOmaiJaeGyoaKdacaGLOaGaayzkaaaaaa@3C7A@

and the biomass concentration *x *at the same time point is

xn=XnVn     (30)
 MathType@MTEF@5@5@+=feaafiart1ev1aaatCvAUfKttLearuWrP9MDH5MBPbIqV92AaeXatLxBI9gBaebbnrfifHhDYfgasaacH8akY=wiFfYdH8Gipec8Eeeu0xXdbba9frFj0=OqFfea0dXdd9vqai=hGuQ8kuc9pgc9s8qqaq=dirpe0xb9q8qiLsFr0=vr0=vr0dc8meaabaqaciaacaGaaeqabaqabeGadaaakeaacqWG4baEdaWgaaWcbaGaemOBa4gabeaakiabg2da9maalaaabaGaemiwaG1aaSbaaSqaaiabd6gaUbqabaaakeaacqWGwbGvdaWgaaWcbaGaemOBa4gabeaaaaGccaWLjaGaaCzcamaabmaabaGaeG4mamJaeGimaadacaGLOaGaayzkaaaaaa@3B29@

The medium feed rate *F*_n _at each time point is

Fn=dSnsf⋅1dt     (31)
 MathType@MTEF@5@5@+=feaafiart1ev1aaatCvAUfKttLearuWrP9MDH5MBPbIqV92AaeXatLxBI9gBaebbnrfifHhDYfgasaacH8akY=wiFfYdH8Gipec8Eeeu0xXdbba9frFj0=OqFfea0dXdd9vqai=hGuQ8kuc9pgc9s8qqaq=dirpe0xb9q8qiLsFr0=vr0=vr0dc8meaabaqaciaacaGaaeqabaqabeGadaaakeaacqWGgbGrdaWgaaWcbaGaemOBa4gabeaakiabg2da9maalaaabaGaemizaqMaem4uam1aaSbaaSqaaiabd6gaUbqabaaakeaacqWGZbWCdaWgaaWcbaGaemOzaygabeaaaaGccqGHflY1daWcaaqaaiabigdaXaqaaiabdsgaKjabdsha0baacaWLjaGaaCzcamaabmaabaGaeG4mamJaeGymaedacaGLOaGaayzkaaaaaa@4244@

These values are used to determine the feed rate profile of the optimized fed batch process.

#### 2. Optimization

The goal of our optimization problem is to find the best values for the specific growth rates *μ*_*n *_and the best value for *dt *(which implies that the total feed period undergoes the optimization process too) such that the volumetric productivity *Q*_*P *_calculated at the point of time *t*_*N*+1 _as

QPN+1=PN+1(t0+tN+1)⋅VN+1     (32)
 MathType@MTEF@5@5@+=feaafiart1ev1aaatCvAUfKttLearuWrP9MDH5MBPbIqV92AaeXatLxBI9gBaebbnrfifHhDYfgasaacH8akY=wiFfYdH8Gipec8Eeeu0xXdbba9frFj0=OqFfea0dXdd9vqai=hGuQ8kuc9pgc9s8qqaq=dirpe0xb9q8qiLsFr0=vr0=vr0dc8meaabaqaciaacaGaaeqabaqabeGadaaakeaacqWGrbqudaWgaaWcbaGaemiuaaLaemOta4Kaey4kaSIaeGymaedabeaakiabg2da9maalaaabaGaemiuaa1aaSbaaSqaaiabd6eaojabgUcaRiabigdaXaqabaaakeaadaqadaqaaiabdsha0naaBaaaleaacqaIWaamaeqaaOGaey4kaSIaemiDaq3aaSbaaSqaaiabd6eaojabgUcaRiabigdaXaqabaaakiaawIcacaGLPaaacqGHflY1cqWGwbGvdaWgaaWcbaGaemOta4Kaey4kaSIaeGymaedabeaaaaGccaWLjaGaaCzcamaabmaabaGaeG4mamJaeGOmaidacaGLOaGaayzkaaaaaa@4C96@

is maximized under the following constraints:

*μ*_min _≤ *μ*_*n *_≤ *μ*_max _for (1 ≤ *n *≤ *N*)     (33)

and

*X*(*t*_*N*+1_) = *X*_max _    (34)

Here *μ*_max _= 0.2 h^-1 ^is the maximum specific growth rate at just below washout in chemostat cultures. Since below *μ *= 0.02 *h*^-1 ^significant product degradation appeared, the lower boundary was set at *μ*_min _= 0.03 *h*^-1^. Also the biomass concentration needs to be limited. The upper limit is mainly defined by the cell separation step, which is practically limited with approximately 100 g*L*^-1 ^dry mass.

##### Remark

Additional constraints may be entered, e.g. the final product concentration may be set at a minimum level.

In the Excel sheet we set *N *= 150. The values *t*_*n*_, *μ*_*n*_, *X_n_*, ... are organized in columns, with each time point *t*_*n*_... a row. The values of *X*_*n*_, *P*_*n*_, *V*_*n*_, ... are calculated from the respective previous row using the equations provided above. The optimization process is performed by the Excel Solver as a black box. It maximizes the final *Q*_*P *_field by varying the *μ *fields within the boundaries and the *dt *field.

The Excel file used for this work is provided as an additional file.

### Analytic approach

To verify the Excel Solver solution, the exact solution of the optimization problem was determined with calculus of variation.

## List of symbols

The symbols, their definitions and units are summarized in table 1.

**Table 1:** List of symbols

Symbol	Definition	unit
c	model parameter	h
*D*	dilution rate	h^-1^
d	axis intercept	g h^-1^
*F*	flow rate	g h^-1^
*F*_L_	flow rate of linear feed	g h^-1^
k	slope	g h^-2^
*k*_q_	Monod constant for *q*_P_	h^-1^
*m*_S_	maintenance coefficient	h^-1^
*p*	product concentration	mg L^-1^
*P*	product mass	mg
*q*_P_	specific product formation rate	mg g^-1 ^h^-1^
*Q*_P_	volumetric productivity	mg L^-1 ^h^-1^
*q*_Pmax_	maximum specific productivity	mg g^-1 ^h^-1^
*S*	substrate mass	g
*s*_f_	substrate concentration	g L^-1^
*s*_*q*_	estimated standard deviation of *q*_P_	mg g^-1 ^h^-1^
*s*_*Y*'_	estimated standard deviation of *Y'*_XS_	-
*t*	time	h
*t*_L_	time of linear feed	h
*T*	total feed time	h
*V*	volume	L
*V*_l_	volume of liquid supernatant	L
*x*	dry biomass concentration	g L^-1^
*X*	dry biomass	g
*Y'*_XS_	observed biomass yield coefficient	-
*Y*_XS_	theoretical biomass yield coefficient	-
		
*Greek symbols*		
*μ*	specific growth rate	h^-1^
*ν*_YDM_	specific volume of biomass	L kg^-1^
*ρ*_f_	density of feed medium	kg L^-1^
*τ*	average residence time	h

## Abbreviations

ddH_2_O double distilled water

Fab2F5 Fab fragment of antibody 2F5

GAP glyceraldehyde-3-phosphate dehydrogenase

OD optical density

YDM yeast dry mass

## Competing interests

The author(s) declare that they have no competing interests.

## Authors' contributions

MM supported designing the model, planned and performed the fermentations, and helped in drafting the manuscript. MK developed the model and the optimization procedure, and prepared part of the manuscript. BG developed the expression strain and supported in fermentations and analytics. DM formulated the project, supported the development of the model and drafted the manuscript. All authors read and approved the final manuscript.

## Supplementary Material

Additional File 1 Model of *P. pastoris *fed batch process for secreted heterologous protein production. This file contains the model as described in Material and Methods, with the optimized values for *μ *and *dt*. By adjusting the specific parameters, other fed batch processes can be modeled and optimized with the Excel Solver. Click here for file
